# 

**Published:** 1893-05

**Authors:** 


### AMERICAN JOURNAL

OF

### DENTAL SCIENCE,

EDITED BY

F. J. S. GORGAS, M. D., D. D. S.,

RICHARD GRADY, M. D., D. D. S.

VOLUME XXVII—THIRD SERIES.

1893-4. SURGEON GENL'S OFFICE 152149. LIBRARY

BALTIMORE:
SNOWDEN & COWMAN.

LONDON:
TRUBNER & CO., 60 PATERNOSTER ROW.

1894.

### Index to Vol. XXVII.—Third Series.

A Study of the Diseases of the Peridental Membrane, having their Origin at or near the Gingival Margin........... 19
About Oxiphosphate .............................. 28
Annual Meeting of the Chicago Dental Society............. 34
Articulating Lower Partials.............................. 46
A Case of Irregularity.................................. 56
A Dental Curiosity...................................... 96
An Act to Regulate the Practice of Dentistry within the Territory of New Mexico.............................. 120
Anæsthetics............................................ 129
A Difficult Case to Control............................ 136
A Misplaced Canine..................................... 137
Abscess of the Antrum of Highmore...................... 140
A Hint................................................. 143
Antiseptic Properties of Tobacco....................... 144
Annual Meeting of the National Association of Dental Faculties............................................ 182
A Simple Holdfast for Porcelain Inlays................. 282
Alabama Dental Association............................. 294
A Brunswick Stew....................................... 325
Amalgam................................................ 458
A Splendid Operation................................... 479
A Plan for Destroying Pulps............................ 480
Aluminum Alloys........................................ 517
Ancient Dentistry...................................... 527
Anæsthetics, and the Physiology of the Heart ... .......... 563
A Few Things to be Remembered.......................... 565
A Bill—Entitled an Act to Repeal and Re-enact with Amendments Article Thirty-two, of the Code of Public General Laws, Entitled "Dentistry"............................ 567
An Interesting Case.................................... 575
Bacteria............................................... 25
Bacteria............................................... 85
Brieflets ............................................. 230
Banding Roots.......................................... 233
Carelessly Swallowed Saliva Mixed with Cocaine........... 95
Cholera Precautions.................................... 133
Campho-phenique........................................ 138
Come to Our Arms ......... ............................. 143

Cements as Root Fillings........ 189
Carcinoma of the Mouth........ 239
Conservative Treatment of the Dental Pulp........ 251
Cocaine in Surgery........ 274
Changes in the University of Maryland........ 281
Chas. Elroy on How to Treat Cocaine Poisoning........ 331
Concerning Various Methods Advocated for Obviating the Necessity of Extracting Devitalized Tooth-pulps........ 356
Cement Filling........ 511
Chloroform Narcosis........ 523
Chicago Dental Society........ 573
Diseased Breath........ 35
Diffuse Cellulitis of the Neck Due to Carious Teeth........ 45
Deaths Under Anæsthetics........ 48
Dr. Chas. B. Atkinson........ 142
Discoloration of Gold Fillings........ 148
Disease in the Antrum........ 154
Dr. Wm. Crenshaw........ 192
Development of Consumption........ 232
Dental Dots Distilled........ 240
Dentistry: Past, Present and Future,........ 241
Development of the Human Tooth........ 351
Dr. Geo. W. McElhaney is Dead........ 377
Deaths under Anæsthetics........ 430
Dr. Henry Snowden........ 433
Dental Dots Distilled........ 479
Dental Education of the Public........ 520
Dentists as Readers........ 524
Dr. A. W. Harlan Retires........ 526
Dentists in Politics........ 528
Extraordinary Surgery........ 37
Extract from a Discussion on Anchorage of Gold Fillings.... 45
Extract from a Discussion on Anchorage of Gold Fillings.... 93
Exemption of Dentists from Jury Duty........ 142
Experiments with Amalgam........ 227
Education........ 281
Eucalyptol........ 336
Empyema of the Antrum........ 339
Etiology of Pyorrhœa Alveolaris........ 552
For Toothache........ 91
Fermentation........ 139
Filling Roots with Gutta Percha Dissolved in Chloroform... 317
Foreign Diplomas in Great Britain........ 332
F. Peterson on Clonic Spasm of the Muscles of Mastication, 333
Fracture of the Jaw........ 370
For the Teeth—Some Excellent Rules to Follow in the Cure of them........ 429

Filling Roots with Chloro-Percha.......... 513
Fitting Bands to Roots.......... 576
Gleanings from the Journals, and Comments.......... 1
Great Eras in Life.......... 68
Germanium.......... 526
Homœopathy in Dental Practice.......... 59
How I Treated an Abscess.......... 288
Hypnotism and Suggestion.......... 367
Hygiene of Hair Dressing and Barber Shops.......... 431
Illinois State Dental Society.......... 88
Immediate Root Filling.......... 123
In Using Cocaine.......... 190
Improvements in Dentistry.......... 289
In Memory of Dr. Wardlaw.......... 375
Inaugural Address.......... 385
Iodide of Potash in Headache.......... 432
Illinois State Dental Society.......... 573
Incidents Connected with the Trial of Professor Webster.... 532
Lockjaw.......... 40
Local Anæsthetic Nostrums.......... 238
Lysol.......... 286
Lubricate Your Disks and Strips.......... 574
Making Metal Dies from Plaster Molds.......... 47
Mutilation of the Teeth by Savages.......... 234
My Method.......... 283
Manipulating Oxyphosphate of Zinc as a Filling-material... 306
Mr. Humby.......... 335
Many a Child in After Years.......... 336
Northwestern University Dental School.......... 88
No American Dental Diplomas Recognized by the General Medical Council of Great Britain.......... 159
New Method of Adjusting Logan Crowns.......... 191
Nitrate of Silver.......... 284
New Remedies and their Application.......... 320
Nervous Exhaustion.......... 329
Neuralgia.......... 426
Number of Dental Colleges and Students.......... 428
Obtunding Dentine.......... 91
Opinion of Dr. Rhein.......... 335
Oral and Facial Surgery.......... 184
On Some Ways of Manipulating the Filling Materials....... 193
Ourselves as Others See Us.......... 364
"Onycophagie".......... 485
Prosthetic Dentistry.......... 481
Porcelain Crown- and Bridge-work.......... 15
Prof. Chapin A. Harris.......... 41

Practical Points........................................ 65
Progress in Therapeutics .............. ............ 134
Period of Infectiousness.... ........ .......... ........ 141
Philosophers and Cranks................................ 190
Presence of Mind in Applying an Antidote................. 191
Prosthetic Dentistry .................................. 276
Pulpless Teeth also Subject to Erosion................... 304
Phagocytosis ............................ ........ 334
Pinus Canadensis............................ ... ....... 432
Prosthetics.............................................. 467
Paralysis Cured by Lancing the Gums on a Little Glrl of Twenty Months.... ........................ 576
Prof. W. H. Eames, D D. S.................................. 572
Removal Bridge or Plate Work........................... 105
Resolutions Adopted by the Chicago Dental Society on the Death of Dr. W. W. Allport................................ 181
Root Treatment....... ..................................... 235
Remarkable Surgical Operations........................... 285
Recent Experiments...................................... 288
Rubber Plates................ ........................... 342
Riggs' Disease—What is it, and can it be Cured ?............ 410
Resolutions Adopted by the Chicago Dental Society on the Death of Dr. W. W. Allport................................ 424
Remarkable Trepanning.................................. 480
Repairing Vulcanite Plates ................................ 573
Sensitive Dentine.......... .............................. 49
Saccharin as an Antiseptic of the Mouth.................... 96
Sarcoma of Lower Jaw—Varicocele—Rarefying Ostitis of Tibia—Deformity of Lower Extremity—Angeio-Sarcoma of Testis.......................................... 97
Soldeiing Aluminum...................................... 144
Spmptomatology of Some Dental Lesions.................. 221
The Teeth During Pregnancy................. ............ 12
The New Mesmerism ...................................... 31
The Dentists at a Disadvantage............................ 43
The Phosphor Necrosis..................................... 82
The Missouri State Dental Association..................... 89
The Other Fellow's Point of View.......................... 90
The Therapeutics of Terraline.............................. 92
The Contraction of Rubber Plates.......... ............... 94
The Lady with the Horse Mane.... ..................... 95
The Conservative Treatment of Teeth by Gold Process...... 113
Treatment of the Ear.................................. ...... 143
Three New Remedial Preparations......................... 145
Things Practical in Dental Practice........... ........... 151
The Great Ordeal........................................ 156

Twenty-fourth Annual Meeting of the California State Dental Society .......... 182
The Progress of Surgery .......... 183
The Use of Anæsthetics in Extracting Teeth .......... 187
The Question of Local Anæsthetic Nostrums .......... 206
Twenty-fourth Annual Session of the Alabama Dental Association .......... 266
The Use of Cocaine as an Anæsthetic .......... 282
Trichloracetic Acid in Pyorrhea .......... 308
The Progress of Dentistry .......... 313
The Bridge-work Fad .......... 330
The Last Straw .......... 336
The Teeth as an Index of Civilization .......... 337
"The Sixth-Year Molar" .......... 345
The Rapid Advance of Dentistry .......... 407
Tin Foil Fillings .......... 416
The Breath .......... 419
The Missouri State Dental Association .......... 424
The Journal in 1893 .......... 425
The Administration of Anæsthetics .......... 430
The Use of Cocaine .......... 431
The Foil for Filling Teeth .......... 439
The Treatment of Teeth During Pregnancy .......... 450
The Teeth and Hair .......... 471
The Maryland Dental Law .......... 475
Teeth Below Medium in Size.—Their Treatment .......... 492
The World's Columbian Dental Congress .......... 499
The Relief of Pain from Diseases of the Dental Pulp and Peridental Membrane .......... 509
The Teeth of Ancient Hawaiians .......... 514
The Discovery of Modern Anæsthesia .......... 522
The New Mineral Carborundum .......... 525
To What Extent and Under What Conditions is the Collar-Crown a Cause of Pericemental Inflammation .......... 529
Treating Infected Root-Canals with Kalium and Natrium .......... 536
The Baltimore College of Dental Surgery .......... 570
University of Buffalo, Dental Department .......... 89
Unnecessary Pain in Dental Operations .......... 110
Unfermented Bread for Invalids .......... 236
U. S. P. 1890.—The Pharmacopœdia of the United States, Seventh Decennial Revision .......... 380
University of Maryland Dental Department .......... 571
Vaccination Ascribed as the Cause of a Death from Lockjaw 463
World's Columbian Dental Congress .......... 34
Welding Gold Crowns .......... 47
What to do in Chloroform Syncope .......... 48

What Produces Caries.... 80
World's Columbian Dental Congress.... 96
World's Columbian Dental Congress.... 175
Women Dentists.... 287
What the Hiccough is.... 314
W. K. Vanderbilt.... 336
Why do Vulcanite Upper Plates Crack?.... 526
What Are the Best Methods for Obtunding Sensibility of the Dentine by Either Local or General Means? Should Arsenic Ever be Used?.... 542

### Communicated.

### WORLD'S COLUMBIAN DENTAL CONGRESS.

TO THE OFFICERS OF DENTAL SOCIETIES IN THE UNITED STATES AND FOREIGN COUNTRIES.

IOWA CITY, IOWA, April 11, 1893.

*American Journal of Dental Science:*

GENTLEMEN:—The Committees on Membership and Registration of the World's Columbian Dental Congress will be saved much trouble and the applicants for membership much vexation if the members of Dental Societies in good standing are furnished with credentials or certificates of membership, so that they may be presented at the desk where intending members apply for their membership cards.

Advanced membership cards will be furnished on application to the Secretary of the General Executive Committee of the Secretary-General of the Congress when the membership fee ($10.00) accompanies the application.

A. O. HUNT,
*Secretary of the General Executive Committee,*
Iowa City, Iowa.

A. W HARLAN, *Sec'y-General of the Congress,*
1000 Masonic Temple, Chicago, Ill.

### ANNUAL MEETING OF THE CHICAGO DENTAL SOCIETY.

At the annual meeting of the Chicago Dental Society, held Tuesday evening, April 4th, 1893, the following officers were elected for the ensuing year:

J. W. Wassall, President; J. H. Woolly, First Vice-

President; Garrett Newkirk, Second Vice-President; L. L. Davis, Recording Secretary; Geo. J. Dennis, Corresponding Secretary; E. D. Swain, Treasurer; J. H. Smyser, Librarian.

BOARD OF DIRECTORS.—Edmund Noyes, J. G. Reid, Geo. H. Cushing.

BOARD OF CENSORS.—E. R. E. Carpenter, D. C. Bacon, W. H. Sale.

GEO. J. DENNIS, *Cor. Sec'y.*

---

### Editorial, Etc.

### To Readers and Correspondents.

Communications intended for publication, and exchange journals and papers, should be addressed to the Editor of The American Journal of Dental Science, No. 845 N. Eutaw St. Baltimore, Md. All letters relating to the business of the Journal, or containing cash remittances, to be directed to Messrs. Snowden & Cowman, No. 9 W. Fayette Street, Baltimore, Md. Articles for publication should be received by the Editor at least two weeks previous to the first of the month on which the number in which it is designed for them to appear, is issued.

### CONTENTS:

Article I.—Gleanings from the Journals, and Comments, By Edwin D. Downs, D. D. S. - - - - - 1

" II.—The Teeth During Pregnancy. - - - - - 12

" III.—Porcelain Crown- and Bridge-Work. By Dr. E. Parmly Brown. - - - - - - 15

" IV.—A Study of the Diseases of the Peridental Membrane, having their Origin at or near the Gingival Margin. By H. A. Kelley, D. M. D. - - - - - 19

" V.—Bacteria. By Dr. N. S. Hoff. - - - - - 25

" VI.—About Oxiphosphate. - - - - - - 28

" VII.—The New Mesmerism. - - - - - - 31

COMMUNICATED.

World's Columbian Dental Congress. - - - - - - 34
Annual Meeting of the Chicago Dental Society. - - - 34

EDITORIAL.

Diseased Breath. - - - - - - - - - - 35

Extraordinary Surgery. - - - - - - - - 37

Lockjaw. - - - - - - - - - - - 40

Prof. Chapin A. Harris. - - - - - - - - 41

MONTHLY SUMMARY.

The Dentists at a Disadvantage. - - - - - - - 43
Diffuse Cellulitis of the Neck Due to Carious Teeth. - - - 45
Extract from a Discussion on Anchorage of Gold Fillings. - - 45
Articulating Lower Partials. - - - - - - - 46
Making Metal Dies from Plaster Molds. - - - - - 47
Welding Gold Crowns. - - - - - - - - - 47
Deaths Under Anæsthetics. - - - - - - - - 48
What to do in Chloroform Syncope. - - - - - - - 48

☞ The American Journal of Dental Science will contain fifty pages of reading matter in each number, making a volume of about Six Hundred pages,

EXCLUSIVE OF ADVERTISEMENTS

